# Low-Dose Atropine Induces Changes in Ocular Biometrics in Myopic Children: Exploring Temporal Changes by Linear Mixed Models and Contribution to Treatment Effect by Mediation Analyses

**DOI:** 10.3390/jcm12041605

**Published:** 2023-02-17

**Authors:** Anders Hvid-Hansen, Nina Jacobsen, Jesper Hjortdal, Flemming Møller, Brice Ozenne, Line Kessel

**Affiliations:** 1 Department of Ophthalmology, Copenhagen University Hospital—Rigshospitalet-Glostrup, DK-2600 Glostrup, Denmark; 2 Department of Clinical Medicine, University of Copenhagen, DK-2200 København N, Denmark; 3 Department of Ophthalmology, Aarhus University Hospital, DK-8200 Aarhus N, Denmark; 4 Department of Ophthalmology, University Hospital of Southern Denmark—Vejle Hospital, DK-7100 Vejle, Denmark; 5 Department of Public Health, Section of Biostatistics, University of Copenhagen, DK-1014 København K, Denmark; 6 Neurobiology Research Unit, Copenhagen University Hospital—Rigshospitalet, DK-2200 København N, Denmark

**Keywords:** myopia, low-dose atropine, ocular biometrics, axial length, spherical equivalent

## Abstract

This study aimed to investigate changes in non-cycloplegic ocular biometrics during the initial six months of treatment with a 0.1% atropine loading dose and 0.01% atropine compared with a placebo and analyze their contribution to the treatment effect on cycloplegic spherical equivalent (SE) progression. The study was based on a randomized, double-masked, placebo-controlled, multicenter trial evaluating a 0.1% atropine six-month loading dose and 0.01% atropine in reducing myopic progression in Danish children. The treatment phase was 24 months, and the washout phase was 12 months. Parameters measured included changes in axial length (AL), anterior chamber depth (ACD), lens thickness (LT), vitreous chamber depth (VCD), and choroidal thickness (ChT), while cycloplegic SE and lens power were calculated. Longitudinal changes and contributions to treatment effects were analyzed using constrained linear mixed models and mediation analyses, respectively. After six months, AL was 0.13 mm shorter (95% confidence interval [CI], −0.18 to −0.07 [adjusted *p* < 0.001]) and 0.06 mm shorter (95% CI, −0.11 to −0.01 [adjusted *p* = 0.060]) with a 0.1% atropine loading dose and 0.01% atropine, respectively, compared to the placebo group. Similar concentration-dependent changes were found with ACD, LT, VCD, ChT, and cycloplegic SE. Although the treatment effects trended toward concentration-dependent responses, only the treatment effect mediated by AL at three months differed significantly between 0.01% atropine and a 0.1% atropine loading dose (adjusted *p* = 0.023). Several ocular biometrics, including AL, ACD, and LT, changed dose-dependently during low-dose atropine treatment. Moreover, the treatment effect of atropine on SE progression was mediated by a subset of ocular biometrics, mainly AL, with trends toward concentration dependency and distributional shifts over time.

## 1. Introduction

Myopia (nearsightedness) is one of the most common eye disorders globally and typically develops during childhood and early adulthood [1]. Myopia occurs when light rays are focused anteriorly to the retina due to excessive refractive power of the cornea or the lens or, more commonly, an increased axial length (AL) of the eye [2]. This refractive imbalance blurs the incoming image and requires optical correction to move the focal point back onto the retina. Importantly, correction with single-vision glasses, contact lenses, or refractive surgery focuses the image but does not reverse the increased risk of sight-threatening eye diseases associated with myopia, particularly high myopia (−6 diopters [D] or more), including retinal detachment, glaucoma, and myopic maculopathy [3,4,5].

The prevalence of myopia is increasing globally, especially in East Asia, and it is estimated that 50% of the world’s population will be myopic by 2050, with 10% having high myopia [4,6]. As a result, there is increasing interest in lowering the lifetime risk of myopia-associated complications by developing interventions to reduce myopia progression in children and teenagers [7]. Interventions currently include pharmacological, optical, and behavioral approaches.

Previous studies have reported disparities between low-dose atropine’s reducing effect on spherical equivalent (SE) progression and AL elongation. Thus, the Atropine for the Treatment of Myopia (ATOM) 2 and the Low-Concentration Atropine for Myopia Progression (LAMP) studies both reported greater reductions in SE progression than AL elongation, and, additionally, ATOM 2 only observed an initial hyperopic shift of +0.3 to +0.4 D at the higher doses of 0.1% and 0.5% atropine [8,9,10]. Recently, the LAMP group further concluded that the myopia-controlling effect of low-dose atropine in terms of SE progression was mainly mediated by reducing AL progression, with no observed changes in other ocular biometrics [10].

We hypothesized that the disparity between AL and SE changes and the hyperopic shift indicate that low-dose atropine acts on multiple ocular biometrics, including AL, to exert its myopia-controlling effect. Since cycloplegia induces transient changes in the biometry of the eye, non-cycloplegic evaluation of these parameters might be needed to detect subtle alterations induced by low-dose atropine [11]. This study investigates changes in non-cycloplegic ocular biometrics and their contribution to the treatment effect on cycloplegic SE progression during the initial six months of treatment with a 0.1% atropine loading dose and 0.01% atropine compared with a placebo.

## 2. Materials and Methods

### 2.1. Participants

We evaluated biometric and refractive changes during the initial six months of follow-up in myopic children enrolled in the Low-dose Atropine for the Prevention of Myopia Progression in Danish Children (APP) study. The study was an investigator-driven, 36-month, equal-allocation, randomized, double-masked, placebo-controlled, multicenter study designed to investigate the efficacy and safety of a 0.1% atropine loading dose and 0.01% atropine-alone eye drops in reducing myopic progression in Danish children. The study was conducted at Copenhagen University Hospital—Rigshospitalet, Aarhus University Hospital, and the University Hospital of Southern Denmark—Vejle Hospital.

The study included children aged 6 to 12 years with myopia (spherical component by cycloplegic autorefraction in at least one eye) of ≤−1 D if age was ≥6 to <9 years, or ≤−2 D if age was ≥9 to ≤12 years, and astigmatism of less than −1.5 D. We excluded those with ocular pathology (e.g., amblyopia, strabismus, keratoconus, retinal dystrophies, and previous eye surgery); systemic diseases (e.g., connective tissue disorders and severe cardiac or respiratory illness); developmental disorders and delays; previous myopia control with atropine, 7-methylxanthine, orthokeratology lenses, and/or other optical interventions; a known allergy to trial medication; and non-compliance with eye examinations.

In phase 1 (treatment phase), the participants were randomized to receive either a 0.1% atropine loading dose for six months followed by 0.01% atropine for 18 months, 0.01% atropine for 24 months, or a placebo for 24 months. The trial medication was administered as one eye drop daily in each eye at bedtime. In phase 2 (washout phase), treatment was stopped, and participants were observed for 12 months. Investigators, study personnel performing the ocular measurements, parents, and participants were masked to the allocation status throughout the entire study period. The study is ongoing; all participants have completed the initial six months, where two doses of atropine were used. An independent researcher additionally masked the statistical analyses by renaming the study ID and the interventional groups.

### 2.2. Examinations

We included biometric and refractive examinations from the initial six months of follow-up. We used IOLMaster 700 (Carl Zeiss Meditec AG, Germany), a swept-source optical coherence tomography (SS-OCT)-based biometer, to measure ocular biometrics on undilated eyes. Ocular biometry included measurements of AL, central corneal thickness (CCT), anterior chamber depth (ACD), and lens thickness (LT). Corneal curvatures, K1 (flattest) and K2 (steepest), and their mean (Km) were measured by Scheimpflug imaging (Pentacam HR, Oculus Optikgeräte GmbH, Germany). 

Choroidal thickness (ChT) was measured by SS-OCT using the DRI OCT Triton on undilated eyes. We used the central 1.0-mm zone of the Early Treatment Diabetic Retinopathy Study (ETDRS) grid obtained from the built-in software, IMAGEnet 6 (Topcon Europe Medical BV, The Netherlands).

Cycloplegic autorefraction was performed with the Retinomax K-plus 3 (Right Mfg. Co. Ltd., Japan) handheld autorefractor. The average of five readings was calculated with a predefined quality cut-off score of ≥7. Cycloplegic autorefraction was performed 30 min after the last of 2 drops of cyclopentolate 1% (Minims Cyclopentolate Hydrochloride 1%, Bausch & Lomb Nordic AB, Sweden) was administered to both eyes at 5 min apart. SE was calculated as spherical power plus half cylinder power. Cycloplegic autorefraction was the only measurement performed under cycloplegia.

Lens power (LP) was calculated using Bennett’s formula, inserting measured (i.e., AL, ACD, and LT) and calculated (i.e., SE, Km, and VCD) values [12]. We used the customized c1 and c2 constants introduced by Rozema et al. and 4/3 as the refractive index of aqueous and vitreous humors [13]. Notably, reported ACD measurements were based on the distance between the corneal endothelium and anterior lens surface, whereas ACD values inserted in Bennett’s formula were measured from the corneal epithelium to the anterior lens surface [13]. Vitreous chamber depth (VCD) was calculated as the difference between AL and the distance from the corneal epithelium to the posterior lens surface (i.e., CCT, ACD, and LT).

### 2.3. Sample Size and Statistical Analysis

The primary endpoint of the APP study was the mean change in AL 36 months after baseline. At the time of planning, there were no valid data on AL elongation in myopic Danish children. Since AL is strongly related to the refraction of the eye, SE was used as a surrogate measure in the power calculation. A two-year myopic progression of −1.2 ± 0.69 D progression has been reported in Asian children [14], while a similar two-year progression of −1.14 ± 0.69 D has been reported in myopic school children wearing single-vision spectacles in Denmark [15]. With a statistical power of 80% at a 5% significance level, we needed a sample size of 21 participants per allocation group to detect a 50% difference in myopia progression after two years. Although we initially planned to enroll 50 children per group to compensate for dropout rates and the unknown effect of low-dose atropine in a European population, the recruitment was terminated before the total number of 150 subjects were enrolled. This decision was based on delays caused by the COVID-19 pandemic, fewer dropouts than anticipated, and a fair margin to the original calculated sample size of 21 children per group. 

Parameters measured on both eyes were averaged for all analyses. We used a constrained linear mixed model (cLMM) to model the evolution of each continuous outcome over time for each allocation group. The mean structure was adjusted for the study site and constrained to be identical between groups at baseline. An unstructured covariance was used to model the residual variance–covariance. Group differences in the change from baseline were tested using Wald statistics, and the uncertainty was quantified based on the observed information and the degrees of freedom using a Satterthwaite approximation. Missing data were implicitly handled in the cLMM by restricted maximum likelihood estimation. 

We then used mediation analyses to quantify how the treatment effect on SE progression, *δ_T_*, was mediated by changes in ocular biometrics [16]. The group differences in the dependent outcome, SE progression, were decomposed into two components: an indirect *δ_I_* and a direct treatment effect *δ_D_*. The first was related to the group differences in other outcomes (AL, LP, ChT, referred to as intermediate outcomes), and the second was independent of the intermediate outcomes. The indirect effect was further decomposed to obtain the contribution of each intermediate outcome: *δ_AL_*, *δ_LP_*, and *δ_ChT_*. The selection of intermediate outcomes was based on existing literature and the intention to minimize the number of variables and interdependency between variables [10,11]. The direct and indirect effects were derived from the parameters of four univariate linear models, one relating the primary outcome to the intermediate outcomes and group (Equation (7)) [16], while the others related each intermediate outcome to the group (Equation (6)) [16]. Parameters were estimated using maximum likelihood on participants with complete data, and statistical inference relied on a normal approximation for the distribution of the Wald statistics.

All hypothesis tests were two-sided. We adjusted for multiple testing using a false discovery rate (FDR) correction across variables and follow-up. An adjusted *p* (adj-*p*) value less than 0.05 was considered statistically significant [17]. 

All statistical analyses were performed using the R statistical software, version 4.1.0 (R Program for Statistical Computing) [18]. The cLMM was estimated using the LMMstar package (R Program for Statistical Computing) [19]. The lava package (R Program for Statistical Computing) was used to perform the mediation analyses [20].

### 2.4. Approvals

The study was registered in the European Union Drug Regulating Authorities Clinical Trials Database (EudraCT: 2018-001286-16) and at www.clinicaltrials.gov (NTC no.: NCT03911271) before initiation. The study was approved by the Committee on Health Research Ethics for the Capital Region of Denmark (reference no.: H-18043987), the Danish Medicines Agency (reference no.: 2018-040088), and the Danish Data Protection Agency (reference no.: P-2022-85). GCP units at Copenhagen University Hospital, Aalborg and Aarhus University Hospitals, and Odense University Hospital monitored the study sites according to the GCP quality standards. We conducted the study following the tenets of the Declaration of Helsinki. Written informed consent was obtained from parents, and verbal assent was obtained from the children.

## 3. Results

A total of 124 subjects were screened for eligibility between May 2019 and April 2021. Ninety-seven participants were randomized to the 0.1% atropine loading dose (*n* = 33), 0.01% atropine (*n* = 32), or placebo (*n* = 32). Mean [standard deviation (SD)] age of participants was 9.4 [1.7] years (Table 1). During the six-month follow-up, one participant from the placebo group withdrew from the study due to parental concerns about potential side effects. Baseline demographics and ocular parameters are presented in Table 1.

### 3.1. Changes in Axial Length, Central Corneal Thickness, and Anterior Chamber Depth

**Figure 1:**
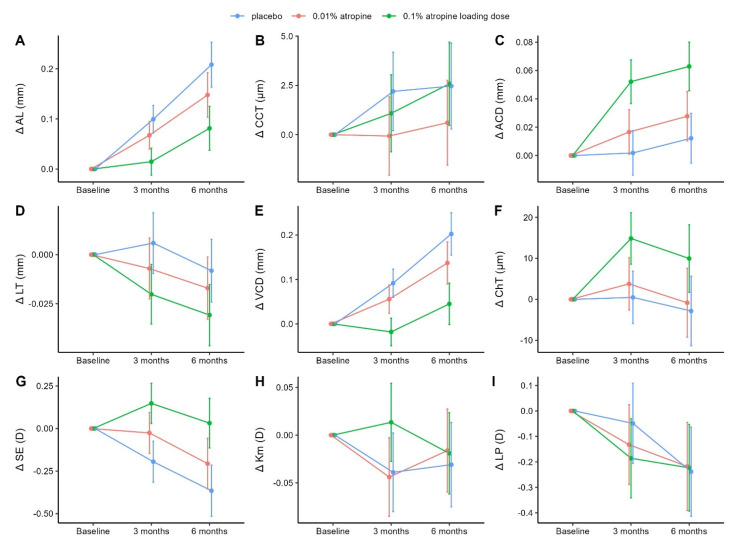
Diagrams showing changes in ocular biometrics and refractive parameters from baseline to six-month visit with placebo (blue), 0.01% atropine (red), and 0.1% atropine loading dose (green). Mean values are indicated by dots, whereas vertical error bars indicate the 95% CIs of the mean changes. Means and CIs were derived from the constrained linear mixed model with inherent baseline adjustment. (**A**), Change in AL in treatment groups over six months. (**B**), Change in CCT in treatment groups over six months. (**C**), Change in ACD in treatment groups over six months. (**D**), Change in LT in treatment groups over six months. (**E**), Change in VCD in treatment groups over six months. (**F**), Change in ChT in treatment groups over six months. (**G**), Change in SE in treatment groups over six months. (**H**), Change in Km in treatment groups over six months. (**I**), Change in LP in treatment groups over six months. **Abbreviations:** Δ = change in; ACD = anterior chamber depth; AL = axial length; CCT = central corneal thickness; ChT = choroidal thickness; CI = confidence interval; D = diopters; Km = mean anterior corneal curvature; LT = lens thickness; LP = lens power; SE = spherical equivalent; VCD = vitreous chamber depth.

AL and ACD showed concentration-dependent changes during the initial six months of follow-up. At six months, the mean change in AL was 0.21 mm (95% confidence interval [CI], 0.16–0.25), 0.15 mm (95% CI, 0.10–0.19), and 0.08 mm (95% confidence interval [CI], 0.04–0.12) in the placebo, 0.01% atropine, and 0.1% atropine loading dose groups, respectively (Figure 1A and Table 2). Differences in AL elongation between the placebo group and interventional groups were −0.06 mm (95% CI, −0.11 to −0.01 [adj-*p* = 0.060]) with 0.01% atropine and −0.13 mm (95% CI, −0.18 to −0.07 [adj-*p* < 0.001]) with the 0.1% atropine loading dose. 

Changes in CCT were small and pairwise comparisons between groups did not show statistically or clinically significant differences (Figure 1B and Table 2).

At six months, the mean increase in ACD was 0.01 mm (95% CI, −0.01 to 0.03) mm, 0.03 mm (95% CI, 0.01–0.05), and 0.06 mm (95% CI, 0.05–0.08) in the placebo, 0.01% atropine, and 0.1% atropine loading dose groups, respectively (Figure 1C and Table 2). Pairwise comparisons showed a statistically significant difference between the 0.1% atropine loading dose group and placebo group of 0.05 mm (95% CI, 0.03–0.07 [adj-*p* < 0.001]), while the difference between the 0.01% atropine group and placebo group of 0.02 mm (95% CI, −0.01 to 0.04 [adj-*p* = 0.224]) was not statistically significant.

### 3.2. Changes in Lens Thickness, Vitreous Chamber Depth, and Choroidal Thickness

These variables also showed a concentration-dependent response. At six months, LT was significantly thinner by −0.02 mm (95% CI, −0.04 to 0.00 [adj-*p* = 0.048]) in the 0.1% loading dose group compared to the placebo group, while the difference in LT between the 0.01% atropine group and placebo group (−0.01 mm [95% CI, −0.03 to 0.01]) was not statistically significant (adj-*p* = 0.464) (Figure 1D and Table 2). 

Over six months, VCD increased in each group, although a temporary shortening of 0.02 mm was observed in the 0.1% atropine loading dose group at three months. At six months, differences in VCD elongation between the placebo group and interventional groups were −0.07 mm (95% CI, −0.12 to −0.01 [adj-*p* = 0.061]) and −0.16 mm (95% CI, −0.21 to −0.10 [adj-*p* < 0.001]) with 0.01% atropine and the 0.1% atropine loading dose, respectively (Figure 1E and Table 2).

ChT increased at 3 months in both interventional groups, especially in the 0.1% atropine loading dose group, and decreased from 3 to 6 months. At six months, pairwise comparisons showed a statistically significant difference between the 0.1% atropine loading dose group and placebo group of 12.8 µm (95% CI, 2.9–22.7 [adj-*p* = 0.039]), while the difference between the 0.01% atropine group and placebo group of 2.0 µm (95% CI, −7.9 to 12.0 [adj-*p* = 0.775]) was not statistically significant (Figure 1F and Table 2).

### 3.3. Changes in Spherical Equivalent, Mean Anterior Corneal Curvature, and Lens Power

Of these parameters, only SE maintained a concentration-dependent response over time. At three months, a hyperopic shift of +0.15 D was noted in the 0.1% atropine loading dose group but not in the 0.01% atropine group. At six months, the mean SE progression was −0.37 D (95% CI, −0.52 to −0.21), −0.21 D (95% CI, −0.35 to −0.06), and +0.03 D (95% CI, −0.11 to 0.18) in the placebo, 0.01% atropine, and 0.1% atropine loading dose groups, respectively (Figure 1G and Table 2). Differences in SE progression between the placebo group and interventional groups were 0.16 D (95% CI, −0.02 to 0.34 [adj-*p* = 0.138]) with 0.01% atropine and 0.40 D (95% CI, 0.22–0.57 [adj-*p* < 0.001]) with the 0.1% atropine loading dose.

Km changes were minimal, and there were no statistically or clinically significant differences between the groups when pairwise comparisons were made (Figure 1H and Table 2).

LP decreased over time in each group, and, despite showing a concentration-dependent response at three months, changes were not significantly different between groups at six months (Figure 1I and Table 2).

### 3.4. Treatment Effect on SE Progression Mediated by Ocular Biometric Parameters

**Figure 2:**
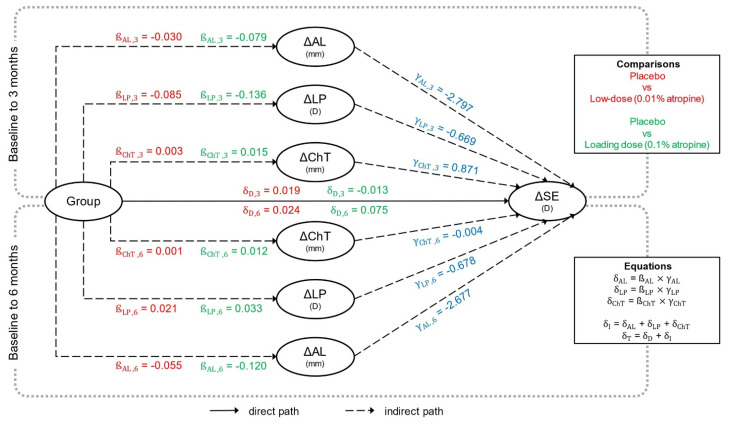
The figure illustrates the results from the mediation analyses. The upper and lower rectangles (marked by dotted lines) report the mediations at 3 and 6 months, respectively. Group-dependent associations (β) are depicted in red (placebo versus low dose) and green (placebo versus loading dose). Group-independent associations (γ) between intermediate outcomes (AL, LP, ChT) and the dependent outcome (SE) are depicted in blue. Group-dependent direct mediations (δ_D_) are drawn in solid lines, whereas indirect mediations are drawn in dotted lines. The equations for determining the mediated effect through an independent variable and the indirect mediation are shown in the equation box. Effect size estimates, differences, and significance levels are reported in Table 3. **Abbreviations:** Δ = change in; AL = axial length; ChT = choroidal thickness; D = diopters; LP = lens power; SE = spherical equivalent.

The contributions to SE progression from direct and indirect treatment effects in the interventional groups compared to the placebo are summarized in Figure 2 and Table 3. Figure 2 presents the group effects on intermediate outcomes, i.e., AL, LP, and ChT (*β* coefficient), the associations between intermediate outcomes and SE progression (*γ* coefficient), and the direct treatment effect on SE progression (*δ_D_*). Thus, to exemplify Figure 2, receiving a 0.1% atropine loading dose was associated with a 0.079 mm shorter AL at 3 months compared with the placebo, and the group-independent *γ* coefficient of *γ_AL,3_* shows that a 1 mm increase in AL was associated with a change in SE of −2.797 D at three months of follow-up.

At three months, the treatment effects on SE progression of 0.01% atropine and the 0.1% atropine loading dose were mainly mediated through changes in AL, with 52.0% and 70.9% of the treatment effect, respectively. LP accounted for 35.1% and 29.1% of the treatment effect with 0.01% atropine and the 0.1% atropine loading dose, respectively, while ChT contributed to 1.4% and 4.1% of the treatment effect comparing 0.01% atropine and the 0.1% atropine loading dose to the placebo, respectively (Table 3). 

At six months, the main treatment effect was still mediated by AL, accounting for more than 85% in both interventional groups compared to the placebo. In contrast, the treatment effect mediated by LP shifted from positive to negative, demonstrating SE progression of −0.014 D (95% CI, −0.151 to 0.123) and −0.023 D (95% CI, −0.160 to 0.115) in the 0.01% atropine and 0.1% atropine loading dose groups, respectively, compared to the placebo group. The treatment effect mediated by ChT ceased almost completely to 0.000 D (95% CI, −0.002 to 0.002) and 0.000 D (95% CI, −0.016 to 0.016) with 0.01% atropine and the 0.1% atropine loading dose, respectively (Table 3). 

Although the treatment effects tended toward concentration-dependent responses, i.e., larger effect sizes with increased atropine concentration, only the difference in mediated treatment effect by AL at three months was significant (adj-*p* = 0.023) between 0.01% atropine and the 0.1% atropine loading dose (Table 3).

## 4. Discussion

We investigated the changes in non-cycloplegic ocular biometric parameters and their contribution to the treatment effect on cycloplegic SE progression during the initial six months of treatment with a 0.1% atropine loading dose and 0.01% atropine compared with a placebo in a randomized, placebo-controlled, multicenter study. We found that several ocular biometrics changed dose-dependently over time. In addition, we found that the treatment effects on SE progression mediated by ocular biometrics also tended toward concentration-dependent responses, which further showed changes over time. To our knowledge, this is the first randomized placebo-controlled trial to demonstrate that ocular biometric parameters change during treatment with low-dose atropine compared to a placebo, and the first to use mediation analyses to report how multiple ocular biometric parameters mediate the treatment effect of low-dose atropine on SE progression.

Previous studies have shown that the effect of low-dose atropine on SE progression is greater than accounted for by the changes in AL and that an initial hyperopic shift can be observed with higher doses [8,9,10]. In agreement with this, we found disproportionate changes between AL elongation (0.08 mm and 0.15 mm) and SE progression (+0.03 D and −0.21 D) after six months in the 0.1% atropine loading dose and 0.01% atropine groups, respectively, when expecting a 1 mm increase in AL to change SE by −2.7 D [21]. Moreover, we found a hyperopic shift of +0.15 D in the 0.1% atropine loading dose group at three months. We believe that the hyperopic shift and the disparity between AL and SE could be explained by the changes that we observed in ACD, LT, VCD, and ChT, which all contribute to the eye’s refraction [13]. These parameters also showed concentration-dependent changes, but the effect was only of significant size, compared to the placebo, at the highest atropine dose (0.1%).

Our findings are supported by observations reported by Gao et al. [11] when examining the changes in ocular biometrics after the instillation of 1% atropine ointment. The study reported an increase in ACD and decreases in LT and VCD under cycloplegia [11]. These changes could be explained by the cycloplegia-induced paralysis of the ciliary muscles, causing the ciliary processes to move outward and backward, shifting the lens–iris apparatus posteriorly, resulting in the deepening of the ACD, thinning of the lens, and shortening of the vitreous chamber [11,22,23]. Other studies reported an increase in subfoveal choroidal thickness during treatment with low-dose atropine, which would further contribute to the shortening of the VCD [24,25]. 

In contrast, biometry measurements performed in cyclopentolate cycloplegia did not show differences in changes in ACD or LP across groups receiving 0.05%, 0.025%, 0.01%, or a placebo in the initial 12 months of the LAMP study [10]. Measures on LT, VCD, and ChT were not obtained; thus, LP was calculated using the Bennett and Rabbetts formula [10]. We speculate that completing the cycloplegic regimen before biometric evaluation might blur the subtle changes induced by different dosages of low-dose atropine, potentially resulting in non-significant differences between groups despite large sample sizes. 

The timing of cycloplegia will also affect the assessment of differences between groups in ocular biometrics’ contributions to SE progression. Recently, the LAMP group concluded that the myopia-controlling effect of low-dose atropine was mainly mediated by the reduction in AL progression, and, additionally, the contribution to SE progression from AL, K, and LP was similar across atropine and placebo groups [10]. These conclusions were made on multivariate linear regressions using adjusted R2 values to describe the proportional variance in SE progression (dependent variable) predicted by cycloplegic measurements on AL, LP, and Km (independent variables) [10]. We performed mediation analyses to directly quantify the treatment effect on SE progression mediated by AL, LP, and ChT. Our findings support those of Li et al. that AL contributed the most to the treatment effect on SE progression but that the other biometrics of the eye also played a role [10]. When evaluating the trends indicated by the estimates from the mediation analyses, the observed decreasing effects of LP and ChT and the increase in direct treatment effect support that atropine affects several components of the eye during treatment. Given that atropine’s myopia-controlling mechanisms are still unknown, a possible explanation for the concentration-dependent effect sizes and their respective changes over time could indicate that the pathways of action depend on the dosing and duration of treatment [26,27]. We encourage researchers within the field of myopia control to include mediation analysis in future reports because effect sizes might be easier to understand than adjusted R2 values.

There are some limitations to our study. First, we only reported the results from six months of follow-up. We chose the time limit as only the first six months of the trial period included two different atropine doses. Second, we did not measure LP directly but used Bennett’s formula with a customized c1 and c2 constant [12,13]. This formula has been shown to be the best calculation of equivalent LP if LT is available [13]. As an alternative, phakometry could be used to obtain the radii of curvature of the anterior and posterior lens surfaces as well as the lens refractive index, but this examination is difficult to perform [13]. Third, the mediation analyses only evaluated the effects mediated by AL, LP, and ChT. The high interdependency between AL, ACD, and VCD did not permit us to run the mediation analyses on these parameters, as the latter was calculated using the former two. We advocate for future studies to also include the anterior and posterior curvatures of the lens, which may improve our understanding of the lenticular changes in relation to changes in ACD and VCD during low-dose atropine treatment. Fourth, the sample size in the APP study was calculated to detect a two-year difference in SE. Although the mediation analyses showed a significant difference in mediated treatment effects by AL, a larger sample size is needed to detect significant differences in other biometrics. 

In conclusion, this randomized, placebo-controlled trial demonstrated that several ocular biometrics, including AL, ACD, and LT, changed dose-dependently during low-dose atropine treatment in children with myopia. In addition, we found that atropine’s treatment effect on SE progression was mediated by a subset of ocular biometrics, mainly AL, with trends toward concentration dependency and distributional shifts over time.

## Figures and Tables

**Table 1:** Baseline demographics and ocular parameters of randomized participants.

		Treatment Groups		
Characteristic	All	Placebo	Low Dose (0.01% Atropine)	Loading Dose (0.1% to 0.01% Atropine)
N (%)	97	32 (33.0)	32 (33.0)	33 (34.0)
Female/male (%)	55/42 (57/43)	18/14 (56.2/43.8)	18/14 (56.2/43.8)	19/14 (57.6/42.4)
Age, mean (SD), years	9.4 (1.7)	9.2 (1.6)	9.4 (1.9)	9.5 (1.5)
AL, mean (SD), mm	24.48 (0.84)	24.41 (0.90)	24.56 (0.78)	24.48 (0.86)
CCT, mean (SD), µm	546.8 (30.3)	546.0 (35.1)	546.4 (25.7)	547.8 (30.3)
ACD, mean (SD), mm	3.31 (0.24)	3.32 (0.27)	3.33 (0.26)	3.28 (0.19)
LT, mean (SD), mm	3.36 (0.17)	3.36 (0.13)	3.36 (0.17)	3.36 (0.20)
VCD, mean (SD), mm	17.27 (0.86)	17.18 (0.89)	17.33 (0.76)	17.30 (0.95)
ChT, mean (SD), µm	248 (66.2)	244 (65.1)	260 (66.7)	240 (67.2)
SE, mean (SD), D	−3.02 (1.27)	−3.07 (1.04)	−2.97 (1.13)	−3.0 (1.59)
Corneal power				
K1, mean (SD), D	43.2 (1.5)	43.4 (1.2)	43.0 (1.2)	43.3 (1.9)
K2, mean (SD), D	44.1 (1.5)	44.3 (1.3)	43.9 (1.2)	44.1 (2.0)
Km, mean (SD), D	43.7 (1.5)	43.8 (1.2)	43.5 (1.2)	43.7 (2.0)
LP, mean (SD), D	22.5 (1.46)	22.6 (1.44)	22.4 (1.28)	22.4 (1.66)

**Abbreviations**: ACD = anterior chamber depth; AL = axial length; CCT = central corneal thickness; ChT = choroidal thickness; D = diopters; K1 = flattest corneal curvature; K2 = steepest corneal curvature; Km = mean anterior corneal curvature; LT = lens thickness; LP = lens power; N = number of participants; SE = spherical equivalent; SD = standard deviation; VCD = vitreous chamber depth.

**Table 2:** Changes in refractive and visual parameters over six months based on constrained linear mixed models.

	Treatment Groups		
Measurement	Placebo †	Low Dose (0.01% Atropine) ‡	Loading Dose (0.1% to 0.01% Atropine) ‡
**AL, mm**			
Baseline §	24.60 (24.35 to 24.86)	―	―
3-month change	0.10 (0.07 to 0.13)	−0.03 (−0.06 to 0.00)	−0.08 (−0.12 to −0.05)
*p* value/adj-*p* value ||	―	0.054/0.114	<0.001/<0.001
6-month change	0.21 (0.16 to 0.25)	−0.06 (−0.11 to −0.01)	−0.13 (−0.18 to −0.07)
*p* value/adj-*p* value ||	―	0.025/0.060	<0.001/<0.001
**CCT, µm**			
Baseline §	551.2 (542.0 to 560.4)	―	―
3-month change	2.2 (0.2 to 4.2)	−2.3 (−4.6 to 0.1)	−1.1 (−3.5 to 1.2)
*p* value/adj-*p* value ||	―	0.060/0.115	0.348/0.464
6-month change	2.5 (0.3 to 4.6)	−1.9 (−4.4 to 0.7)	0.1 (−2.4 to 2.7)
*p* value/adj-*p* value ||	―	0.152/0.225	0.928/0.928
**ACD, mm**			
Baseline §	3.30 (3.23 to 3.37)	―	―
3-month change	0.00 (−0.01 to 0.02)	0.01 (0.00 to 0.03)	0.05 (0.03 to 0.07)
*p* value/adj-*p* value ||	―	0.117/0.201	<0.001/<0.001
6-month change	0.01 (−0.01 to 0.03)	0.02 (−0.01 to 0.04)	0.05 (0.03 to 0.07)
*p* value/adj-*p* value ||	―	0.137/0.224	<0.001/<0.001
**LT, mm**			
Baseline §	3.33 (3.28 to 3.38)	―	―
3-month change	0.01 (−0.01 to 0.02)	−0.01 (−0.03 to 0.01)	−0.03 (−0.04 to −0.01)
*p* value/adj-*p* value ||	―	0.156/0.225	0.005/0.016
6-month change	−0.01 (−0.02 to 0.01)	−0.01 (−0.03 to 0.01)	−0.02 (−0.04 to 0.00)
*p* value/adj-*p* value ||	―	0.338/0.464	0.016/0.048
**VCD, mm**			
Baseline §	17.43 (17.17 to 17.69)	―	―
3-month change	0.09 (0.06 to 0.12)	−0.04 (−0.07 to 0.00)	−0.11 (−0.15 to −0.07)
*p* value/adj-*p* value ||	―	0.061/0.115	<0.001/<0.001
6-month change	0.20 (0.15 to 0.25)	−0.07 (−0.12 to −0.01)	−0.16 (−0.21 to −0.10)
*p* value/adj-*p* value ||	―	0.022/0.058	<0.001/<0.001
**ChT, µm**			
Baseline §	242.1 (221.9 to 262.3)	―	―
3-month change	0.5 (−5.9 to 6.9)	3.3 (−4.3 to 10.9)	14.4 (6.8 to 21.9)
*p* value/adj-*p* value ||	―	0.392/0.486	<0.001/0.001
6-month change	−2.8 (−11.3 to 5.6)	2.0 (−7.9 to 12.0)	12.8 (2.9 to 22.7)
*p* value/adj-*p* value ||	―	0.689/0.775	0.012/0.039
**SE, D**			
Baseline §	−2.99 (−3.37 to −2.60)	―	―
3-month change	−0.20 (−0.32 to −0.07)	0.17 (0.03 to 0.31)	0.34 (0.20 to 0.48)
*p* value/adj-*p* value ||	―	0.021/0.057	<0.001/<0.001
6-month change	−0.37 (−0.52 to −0.21)	0.16 (−0.02 to 0.34)	0.40 (0.22 to 0.57)
*p* value/adj-*p* value ||	―	0.077/0.138	<0.001/<0.001
**Km, D**			
Baseline §	43.46 (43.02 to 43.91)	―	―
3-month change	−0.04 (−0.08 to 0.00)	0.00 (−0.05 to 0.04)	0.05 (0.00 to 0.10)
*p* value/adj-*p* value ||	―	0.840/0.893	0.036/0.080
6-month change	−0.03 (−0.08 to 0.01)	0.01 (−0.04 to 0.07)	0.01 (−0.04 to 0.06)
*p* value/adj-*p* value ||	―	0.574/0.689	0.648/0.753
**LP, D**			
Baseline §	22.24 (21.80 to 22.67)	―	―
3-month change	−0.05 (−0.21 to 0.11)	−0.08 (−0.27 to 0.10)	−0.14 (−0.32 to 0.05)
*p* value/adj-*p* value ||	―	0.376/0.483	0.146/0.225
6-month change	−0.24 (−0.41 to −0.06)	0.02 (−0.19 to 0.23)	0.02 (−0.19 to 0.22)
*p* value/adj-*p* value ||	―	0.843/0.893	0.883/0.909

**Notes:** Changes in ocular biometrics and refractive parameters from baseline to six-month visit. All estimates were determined using the constrained linear mixed models with inherent baseline adjustments. Changes in the placebo group are presented as mean change from baseline (95% CI). Changes in the 0.01% atropine and 0.1% atropine loading dose groups are presented as differences from the placebo group as mean (95% CI). **Footnotes:** †, presented as mean change from baseline (95% CI); ‡, presented as difference from the placebo group as mean (95% CI); §, baseline value was the same for all groups; ||, adjusted for false discovery rate across parameters and follow-up. **Abbreviations:** ACD = anterior chamber depth; adj-*p* = adjusted *p*; AL = axial length; CCT = central corneal thickness; ChT = choroidal thickness; CI = confidence interval; D = diopters; Km = mean anterior corneal curvature; LT = lens thickness; LP = lens power; SE = spherical equivalent; VCD = vitreous chamber depth.

**Table 3:** Mediation analyses.

	Estimates						Differences		Significance
	Placebo vs. Low Dose (0.01% Atropine)			Placebo vs. Loading Dose (0.1% to 0.01% Atropine)			Effect Size		Differences in Effect Size
	Estim.	(95% CI)	% †	Estim.	(95% CI)	% †	Estim.	(95% CI)	*p*/adj-*p* ‡
**3 months (N = 92)**									
Direct (δ_D,3_)	0.02	(−0.04; +0.08)	11.5	−0.01	(−0.08; +0.06)	−4.1	−0.03	(−0.10; +0.03)	0.336/0.537
Indirect (δ_I,3_)	0.14	(−0.01; +0.29)	88.5	0.33	(0.17; 0.48)	104.1	0.18	(0.03; 0.34)	0.020/―
AL, mm (δ_AL,3_)	0.08	(−0.01; +0.17)	52.0	0.22	(0.13; 0.32)	70.9	0.14	(0.05; 0.23)	0.003/0.023
LP, D (δ_LP,3_)	0.06	(−0.07; +0.18)	35.1	0.09	(−0.03; +0.21)	29.1	0.03	(−0.09; +0.16)	0.583/0.777
ChT, mm (δ_ChT,3_)	0.00	(−0.01; +0.01)	1.4	0.01	(−0.01; +0.04)	4.1	0.01	(−0.01; +0.03)	0.306/0.537
Total (δ_T,3_)	0.16	(0.00; 0.32)	100.0	0.31	(0.15; 0.48)	100.0	0.15	(−0.01; +0.31)	0.068/―
**6 months (N = 94)**									
Direct (δ_D,6_)	0.02	(−0.04; +0.09)	15.2	0.08	(0.01; 0.14)	20.1	0.05	(−0.01; +0.12)	0.121/0.322
Indirect (δ_I,6_)	0.13	(−0.06; +0.33)	84.8	0.30	(0.10; 0.50)	79.9	0.16	(−0.03; +0.36)	0.101/―
AL, mm (δ_AL,6_)	0.15	(0.01; +0.29)	93.7	0.32	(0.18; 0.46)	86.0	0.17	(0.03; 0.31)	0.015/0.061
LP, D (δ_LP,6_)	−0.01	(−0.15; +0.12)	−8.9	−0.02	(−0.16; +0.12)	−6.1	−0.01	(−0.15; +0.13)	0.901/0.995
ChT, mm (δ_ChT,6_)	0.00	(−0.00; +0.00)	0.0	0.00	(−0.02; +0.02)	0.0	0.00	(−0.01; 0.01)	0.995/0.995
Total (δ_T,6_)	0.16	(−0.05; +0.36)	100.0	0.37	(0.17; 0.58)	100.0	0.22	(0.01; 0.42)	0.039/―

**Notes:** The mediation analyses show how changes in ocular biometrics mediated the treatment effect on SE progression at three and six months of follow-up. The group difference in the dependent outcome, SE progression, is decomposed into two components: a direct (δ_D_) and an indirect (δ_I_) treatment effect. The indirect effect (δ_I_) is further decomposed to obtain the contribution of each intermediate outcome: δ_AL_, δ_LP_, and δ_ChT_. Mediation analyses were performed on participants with complete data. See Figure 2 for a graphical representation. **Footnotes:** †, presented as a percentage of the total effect (δ_T_); ‡, adjusted for false discovery rate (FDR) across variables and follow-up. Adjustments are not performed on ‘Indirect’ and ‘Total’ variables as these denote sums of other variables. **Abbreviations:** adj-*p* = adjusted *p*; AL = axial length; ChT = choroidal thickness; CI = confidence interval; D = diopters; Estim. = Estimate; FDR = false discovery rate; LP = lens power; N = number of participants.

## Data Availability

Anonymized datasets are available from the corresponding author on reasonable request.
